# DAILY SUPPORT EXCHANGES WITH FRIENDS AND PSYCHOLOGICAL WELL-BEING IN LATE LIFE

**DOI:** 10.1093/geroni/igad104.1504

**Published:** 2023-12-21

**Authors:** Yee To Ng, William Chopik, Karen Fingerman

**Affiliations:** Institution of Social Researach, Ann Arbor, Michigan, United States; Michigan State University, East Lansing, Michigan, United States; The University of Texas at Austin, Austin, Texas, United States

## Abstract

Support exchanges are crucial to older adults’ well-being. Functionalist and identity theories suggest that individuals engage in support exchanges with social ties that are consistent with their identities/roles to maximize their well-being. This study examined support exchanges with friends in daily life and tested the link between support exchanges with friends and daily well-being, particularly how this link varied between married and unmarried older adults. Adults aged 65+ (N = 292, Mean age = 73.72, 60% married) provided information about their demographics and social partners. Across five days, they completed surveys about support exchanges with friends (i.e., receiving or giving advice, practical support, and emotional support, respectively) and daily life satisfaction at the end of each day. Multilevel logistic models revealed that unmarried older adults were more likely to receive all types of support from friends and also more likely to provide advice and emotional support (non-tangible support) to friends than married older adults. Support provision was unrelated to life satisfaction. On days when older adults received advice from friends, they reported increased life satisfaction than on days that they did not (within-person association). This within-person association was stronger among unmarried than married older adults. Patterns of findings remained unchanged after adjusting for support exchanges with other social ties. Findings highlight the role of marital status in older adults’ daily support exchanges. Unmarried older adults may compensate for the lack of a spouse with friends and are more reactive to daily support exchanges than married older adults.

